# Interplay of Rad51 with NF-κB Pathway Stimulates Expression of HIV-1

**DOI:** 10.1371/journal.pone.0098304

**Published:** 2014-05-21

**Authors:** Rafal Kaminski, Hassen S. Wollebo, Prasun K. Datta, Martyn K. White, Shohreh Amini, Kamel Khalili

**Affiliations:** 1 Department of Neuroscience, Center for Neurovirology, Temple University School of Medicine, Philadelphia, Pennsylvania, United States of America; 2 Department of Biology, College of Science and Technology, Temple University, Philadelphia, Pennsylvania, United States of America; George Mason University, United States of America

## Abstract

Transcription from the HIV-1 promoter is controlled by a series of ubiquitous and inducible cellular proteins with the ability to enter the nucleus and interact with the promoter. A DNA sequence spanning nucleotides −120 to −80, which supports the association of the inducible NF-κB transcription factor, has received much attention. Here we demonstrate that the interplay between Rad51, a key regulator of the homologous recombination pathway of DNA repair and whose level is induced upon HIV-1 infection, with the NF-κB pathway, augments transcription of the viral promoter. Evidently, stimulation of the NF-κB pathway by PMA and/or TSA promotes association of Rad51 with the LTR DNA sequence and that the p65 subunit of NF-κB is important for this event. Our results also demonstrate that, similar to p65, Rad51 utilizes the NF-κB pathway to position itself in the nucleus as ectopic expression of an IκB mutant impedes its nuclear appearance and transcriptional activity upon the HIV-1 LTR. Treatment of peripheral blood mononuclear cells with small molecules that inhibit Rad51 activity results in greater than 50% decrease in the HIV-1 infection of cells. These observations provide evidence for the involvement of DNA repair factors in control of HIV-1 gene activation and offer a new avenue for the development of anti-viral therapeutics that affect viral gene transcription in latently infected cells.

## Introduction

Similar to other lentiviruses, the long terminal repeat (LTR) of HIV-1 is divided into three functional regions, including core promoter, enhancer and modulatory domains. The core promoter, positioned immediately upstream from the transcription start site, contains A/T and G/C-rich elements that support assembly of the basal RNA polymerase II complex and association with a ubiquitous transcription factor, SP family, respectively [1]. The enhancer region, which is flanked by the core and modulatory domains, has a characteristic primary DNA structure for interaction with the NF-κB inducible transcription factors and its affiliated proteins [2]. The modulatory domain provides binding sites for a plethora of positive and negative cellular factors including C/EBP, ATF/CREB, LEF-1, NFAT, and others [3]. The enhancer domain of the HIV-1 LTR has captured much attention due to its critical role in the stimulation of viral gene expression during the course of a productive infection cycle and its participation in many biological events that dictate the state of virus latency and reactivation [4]. The interaction of NF-κB subunits, most notably p65, has been repeatedly demonstrated with the two copies of the κB binding site within the enhancer element of HIV-1 in various cell types [2], [5]–[7]. The NF-κB family of proteins including p65 is commonly maintained in an inactive form through its sequestration in the cytoplasm by binding to its inhibitor, IκB [8]. Under certain environmental stress, IκB is rapidly phosphorylated at the specific amino acid residues by IκB kinase complexes, IκKα, -β, and -γ, and upon ubiquitination proteolytically degraded and release NF-κB p65 [9]–[11]. Free NF-κB p65, in turn, translocates to the nucleus and upon binding to its target DNA sequence, stimulates its transcription. Several studies have also demonstrated that physical and functional interaction of NF-κB p65 with other factors, cellular and viral, impacts transcriptional activity of NF-κB p65 and its partners. These include C/EBP family members [12], glucocorticoid receptor [13], TATA-binding protein (TBP) and TFIIB [14], YB-1 [15], estrogen receptor-alpha [16], NFBP [17] and HIV-1 transactivator Tat [18].

Our earlier studies suggest that HIV-1 infection of microglia leads to the induction of Rad51 [19]. Rad51 is a key regulator of the homologous recombination pathway of DNA repair whose primary function is believed to be repair of double-stranded breaks and interstrand crosslinks, and maintenance and rescue of DNA replication forks and telomerases [20], [21]. The key step in homologous recombination is the formation of functional Rad51 nucleofilament that requires participation of other proteins such as BRCA2, Rad52 and others [22], [23]. Here we demonstrate that Rad51 has the ability to stimulate transcription of the HIV-1 promoter and associates with the viral DNA sequence spanning the κB motif. Moreover, we demonstrate that inhibition of Rad51 diminishes replication of HIV-1 in peripheral blood mononuclear cells. These observations ascribe a new role for Rad51 in stimulating transcription of the eukaryotic promoter via the NF-κB pathway.

## Results

**Figure 1:**
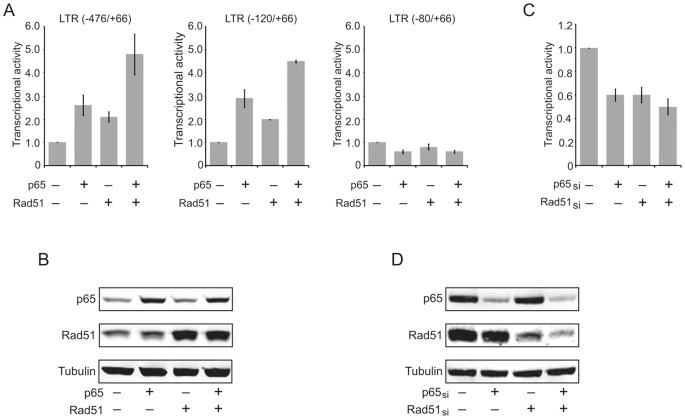
Activation of the HIV-1 LTR by Rad51 and NF-κB p65 requires the κB motif. **(A)** Primary cultures of human astrocytes were transfected with luciferase reporter plasmids pGL3-LTR encompassing various segments of the LTR (as indicated), either alone or together with plasmids expressing NF-κB p65 and/or Rad51. The total amount of DNA was equalized with relevant plasmid vector DNA. **(B)** Western blot of protein extracts from the cells shown in Panel A showing NF-κB p65 and Rad51 levels in each sample. α-Tubulin served as a protein loading control. **(C)** Primary cultures of human astrocytes were transfected with NF-κB p65 siRNA and Rad51 siRNA alone or together. The total amount of siRNA was equalized with non-target siRNA. After 48 hours, cells were lysed and luciferase assays performed. **(D)** Western blot analysis illustrating the levels of NF-κB p65 and Rad51 in cells treated with various siRNAs as indicated. Transcriptional activity represents fold effect in which controls (NF-κB p65 and Rad51) were set at 1.0.

We examined the effect of Rad51 on transcription of HIV-1 in primary cultures of human fetal astrocytes and demonstrated that ectopic expression of Rad51, either alone or in concert with the p65 subunit of NF-κB, elevates the activity of the full-length LTR promoter spanning nucleotides −476 to +66 and its deletion mutant −120 to +66 (P≤.05 for both), but not mutant −80 to +66, which lacks the binding site for the inducible NF-κB transcription factor (Fig. 1A). In a complementary approach, we evaluated the activity of the LTR in cells whose intracellular levels of Rad51 and NF-κB p65 were reduced by Rad51 or p65-specific siRNAs. As seen in Fig. 1C, silencing of NF-κB p65 or Rad51 decreased the level of LTR transcription in primary cultures of astrocytes (P≤.05 for both). Figure 1 also illustrates the levels of NF-κB p65 and Rad51 along with a housekeeping protein, α-tubulin, in cells with overexpression (Panel B) or suppression (Panel D) of these proteins in transfected cells.

**Figure 2:**
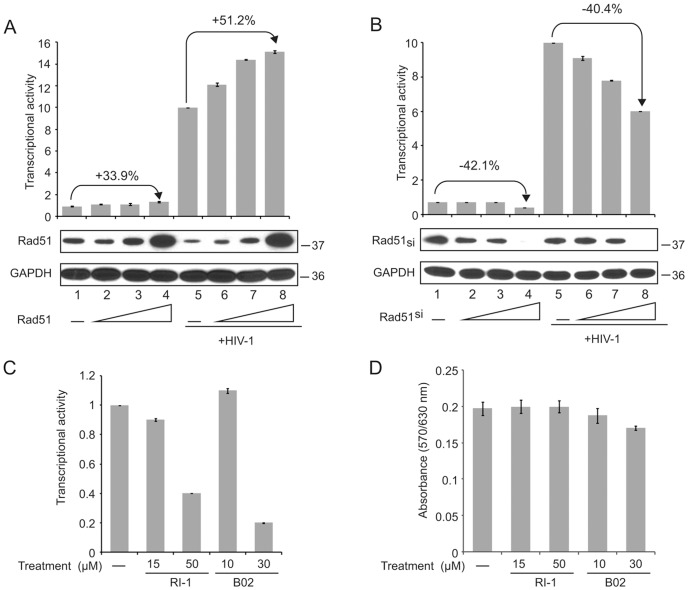
Rad51 stimulates expression of integrated copies of HIV-1. **(A)** TZM-bl cells multiple endogenous integrated copies of LTR-luciferase were transfected with 0, 3, 10 and 30 ng of pCMV (lanes 1–4 or 5–8, respectively). After 24 hours, cells either remained unchanged or infected with HIV-1 and 48 hours later cells were harvested for luciferase assay for promoter activity and Western blot for measuring Rad51 level. **(B)** TZM-bl cells were treated as above except that plasmid expressing siRad51 was used to decrease expression of endogenous Rad51. Luciferase activity and Rad51 Western blots are shown. **(C)** Primary human fetal astrocytes were transfected with luciferase reporter plasmid treated with RI-1 or B02 in various concentrations after 24 hours and luciferase activity was determined after 48 hours. **(D)** MTT assay showing cell viability upon treatment under the same conditions used in Panel C.

Next, we used TZM-bl, a cell line which contains multiple endogenous integrated copies of the DNA sequence corresponding to the HIV-1 LTR in fusion with luciferase gene, to evaluate the impact of Rad51 upon transcription of the viral promoter. Results showed that the activity of the integrated LTR DNA sequence of HIV-1 partially relies on the level of Rad51 in the cells. As seen in Figure 2, overproduction of Rad51 by CMV-Rad51 plasmid (Panel A, compare lane 1 to lane 4), or suppression of this protein by Rad51 siRNA (Panel B, compare lane 1 to lane 4), respectively increased (33.9%) or decreased (42.1%) viral gene transcription (P≤.05 for both). Infection of these cells with HIV-1, which results in the expression of the viral transactivator, Tat, significantly enhances the level of transcription from the integrated copy of the LTR (Fig. 2, Panels A and B, compare lanes 1 and 5, P≤.05). Again, increasing the level of Rad51 expression caused enhancement in the transcription of the LTR in the infected cells (Panel A, lanes 5–8, P≤.05), whereas silencing of Rad51 noticeably diminished LTR promoter activity (Panel B, lanes 5–8, P≤.05). Interestingly, the level of Rad51 was not induced or even slightly reduced by HIV-1 infection in these cells in the absence of Rad51 expression plasmid or siRNA transfection (compare 2A, lane 1 to lane 5 and 2B, lane 1 to lane 5). This is likely because they are a highly transformed HeLa cell derivative and Rad51 levels are already much induced due to many of the cells being in S-phase where Rad51 is maximally expressed [24]. In the next set of studies, we examined the impact of two recently developed small molecules RI-1 and B02 [25], [26] that inhibit Rad51 function upon HIV-1 LTR transcription in human primary astrocytes. RI-1 covalently binds to RAD51 at Cys319 and irreversibly destabilizes a protein-protein interface that is essential for filament formation and recombinase activity while B02 directly interacts with RAD51 (Kd = 5.6 µM), and disrupts its binding to DNA and nucleoprotein filament formation. As seen in Figure 2 (Panel C), incubation of cells with 15 and 50 µM RI-1 suppresses LTR promoter activity (P≤.05). While incubation of cells with 10 µM of B02 showed no effect on LTR transcription at the higher concentration, i.e. 30 µM, the viral promoter activity was drastically diminished in the presence of this inhibitor (P≤.05). MTT assay (Fig. 2D) showed that there was no change in the viability of the cells at the concentrations of inhibitors that were used except for a small decline (15%) at 30 µM B02. Together, these observations indicate that Rad51 controls the level of transcription from the HIV-1 promoter and that a region corresponding to κB motif is important for this regulatory event.

**Figure 3:**
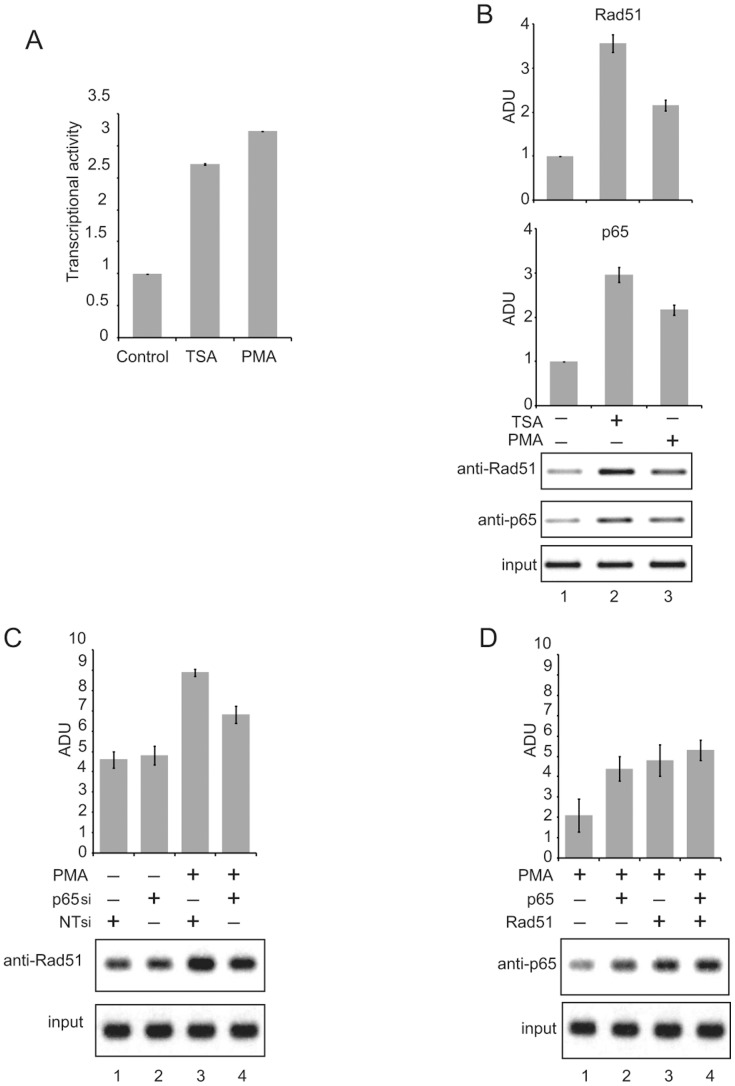
Recruitment of Rad51 to an integrated copy of the HIV-1 LTR and activation of its transcription. **(A)** TZM-bl cells were treated with TSA or PMA and the level of promoter activity was determined after 36 hours by luciferase assay. **(B)** ChIP analysis of extracts from TZM-bl cells treated with TSA and PMA illustrating the presence of LTR DNA corresponding to the κB motif in immunocomplexes pulled down by anti-Rad51 and anti-p65 antibodies. The bar graphs illustrate quantitative analyses of the intensity of bands corresponding to LTR DNA detected in the complex pulled down by anti-Rad51 and anti-p65 antibodies. The intensity of the input band was used for normalizing the data of each. **(C)** ChIP analysis for detection of Rad51 association with endogenous LTR DNA in TZM-bl cells, untreated or treated with PMA, after transfection with plasmids expressing non-target (NT) siRNA or NF-κB p65-specific siRNA. The bar graph illustrates quantitative analysis of the intensity of bands corresponding to LTR DNA detected in the complex pulled down by anti-Rad51 antibody. The intensity of the input band was used for normalizing the data. **(D)** ChIP analysis demonstrating the interaction of NF-κB p65 with integrated copies of the LTR under various conditions as indicated. ADU (Absorption density units) were set at the arbitrary unit of one. Ten represents the intensity of the bands obtained from ChIP assay.

In the next experiments, we investigated the in vivo association of Rad51 and NF-κB p65 with the HIV-1 LTR DNA sequence and the activity of the viral promoter upon induction of the NF-κB pathway by either PMA or the histone deacetylase inhibitor TSA in TZM-bl cells. Treatment of the cells with either PMA or TSA enhanced the level of endogenous HIV-1 promoter (Fig. 3A, P≤.05 for both). Under identical conditions, examination of Rad51 and NF-κB p65 interaction with LTR DNA by ChIP assay showed a substantial increase in the level of Rad51 interaction with viral DNA (Fig. 3B, P≤.05 for both), as expected, this treatment promoted p65 association with LTR DNA (P≤.05 for both). To further assess the cooperativity between Rad51 and NF-κB pathways upon the HIV-1 LTR, we examined the level of interaction of Rad51 with the LTR in cells in which the NF-κB pathway was induced by PMA treatment and the level of NF-κB p65 is diminished by p65-specific siRNA in PMA-treated cells (P≤.05). Results from ChIP assays showed that transfection of cells with NF-κB p65 siRNA partially diminished the interaction of Rad51 with LTR DNA (Fig. 3C, compare bars 3 and 4, P≤.05), suggesting that NF-κB p65 may contribute to Rad51 interaction with the LTR. Similar to those shown in Figure 1 (Panel D), the level of NF-κB p65 in cells in the absence and presence of p65 siRNA was monitored to ensure the efficiency of the siRNA in reducing p65 levels in the cells (not shown). Ectopic expression of p65 or Rad51 in PMA-treated cells further elevated NF-κB p65 association with DNA to a relatively small extent (Fig. 3D, P≤.05).

**Figure 4:**
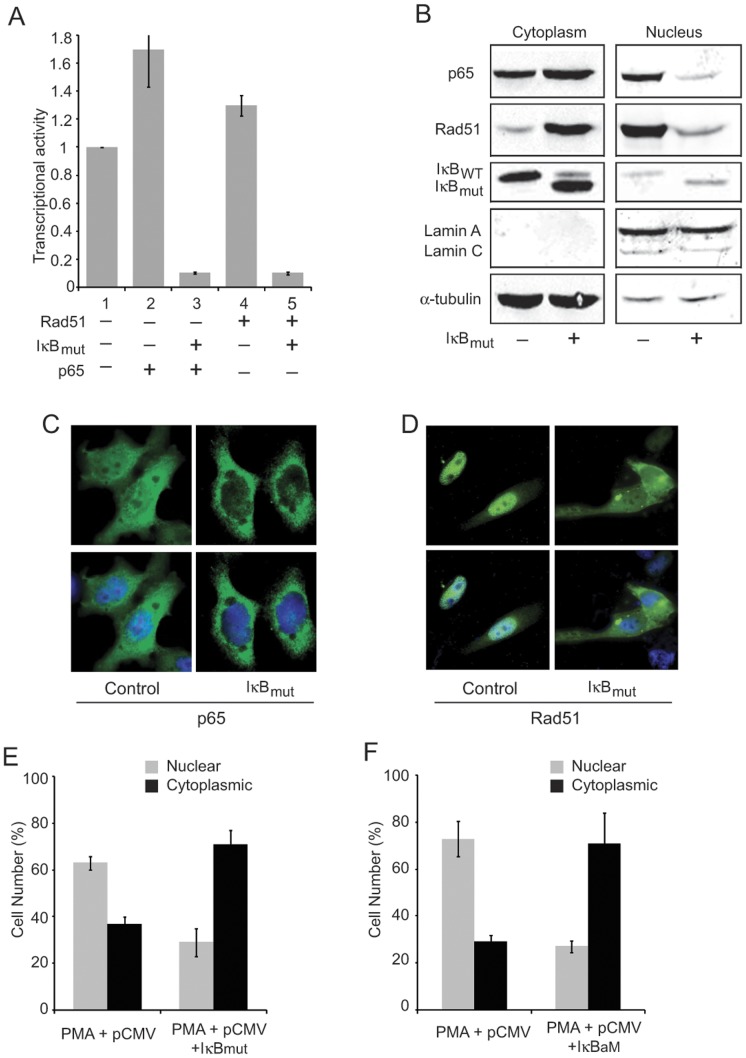
Impact of NF-κB pathway on Rad51 activation of the LTR. **(A)** Primary cultures of astrocytes were transfected with LTR-luciferase reporter constructs along with plasmids expressing Rad51, NF-κB p65 and IκB_mut._in various combinations as indicated. Luciferase activity was determined after 36 hours and fold changes are illustrated. **(B)** Cytoplasmic or nuclear protein fractions from extracts of TZM-bl cells transfected with plasmids expressing NF-κB p65, Rad51 and IκB_mut_. were analyzed for p65 and Rad51 by Western blot. Lamin and α-tubulin were used to measure the integrity of the protein extracts. **(C)** Subcellular localization of p65 in TZM-bl cells transfected with pCMV-p65, pCMV-myc-Rad51 and either with pCMV (control) plasmid or a plasmid expressing IκB_mut_ and treated with PMA. **(D)** Subcellular localization of myc-Rad51 in TZM-bl cells transfected with pCMV-p65, pCMV-myc-Rad51 and either control or IκB_mut_, and treated with PMA. **(E)** Quantification of the image shown in Panel C was performed by scoring 50 cells. **(F)** Quantification of the image shown in Panel D was performed by scoring 50 cells.

Our observation on the ability of Rad51 to associate with the HIV-1 LTR and stimulate its transcriptional activity, an event that requires the cooperation of NF-κB p65, prompted us to further investigate the impact of NF-κB pathways on this activity. As such, we examined the possible role of IκB, a known regulator of NF-κB, which upon its phosphorylation by IKK promotes nuclear entry of NF-κB p65, in Rad51 activation of the LTR. As shown in Figure 4A, we found that ectopic expression of a dominant negative mutant of IκB (IκB_mut_), which contains mutations in the phosphorylation sites for IKK at serine residues 32 and 36, impairs transcriptional activation of the LTR by Rad51 (P≤.05) and, as anticipated, by NF-κB p65 (P≤.05). We also examined the level of Rad51 in the cytoplasmic and nuclear protein fractions of cells expressing IκB_mut_. As seen in Fig. 4B, expression of IκB_mut_ significantly deceased the nuclear presence of Rad51 and at the same time increased its levels in the cytoplasm. As expected, IκB_mut_ significantly diminished nuclear appearance of NF-κB p65. Immunofluorescence studies confirmed these data (Figs. 4C–F). These observations suggest that, similar to p65, Rad51 employs the NF-κB pathway to enter the nucleus and through association with the LTR DNA sequence and cooperation with NF-κB p65 enhances transcription of the HIV-1 genome.

**Figure 5:**
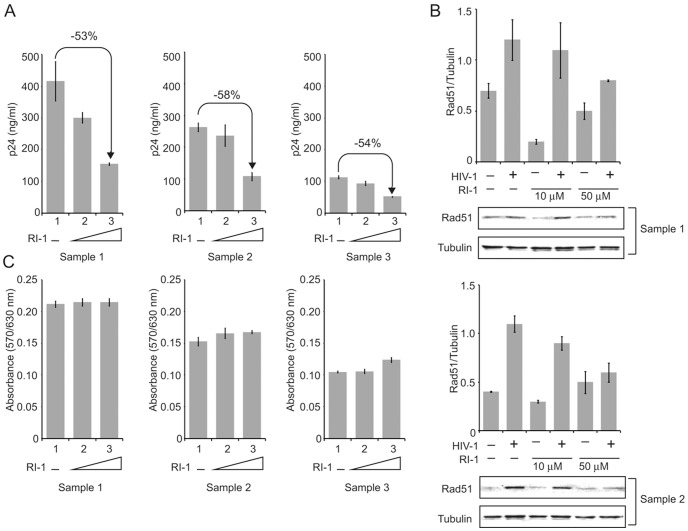
Inhibition of Rad51 function represses HIV-1 infection. **(A)** Top: PBMCs isolated from 3 different donors were infected with HIV-1 SF_162_ and incubated in the presence of 10 or 50 µM of Rad51 inhibitor (RI-1) for 3 days. Infection was quantified by examining the level of virus in the supernatants from the infected cells using p24 Gag ELISA. **(B)** Western blot analysis of protein extracts from the infected cells using anti-Rad51 and anti-α-tubulin antibody. The ratio of the intensity of the Rad51 and α-tubulin in each sample is shown. **(C)** MTT assay showing cell viability upon treatment under the same conditions used in Panel A.

Next, we evaluated the importance of Rad51 in infection of primary culture of human peripheral blood mononuclear cells (PBMCs) by HIV-1. In this experiment, we utilized an inhibitor of Rad51, RI-1, which is a chemically synthesized small molecule that covalently binds to Rad51 and prevents its function [25]. Results from p24 assays demonstrated that treatment of PBMCs prepared from three different donors with RI-1 significantly decreased (approximately 50%) the level of HIV-1 infection of these cells (Fig. 5A, P≤.05 for all samples). Examination of Rad51 in the uninfected and HIV-1 infected samples demonstrated that while infection of the PBMCs with HIV-1 increased expression of Rad51 in the cells (P≤.05), RI-1 was able to significantly reduce Rad51 levels (Fig. 5B, P≤.05). Of note, under the concentrations where RI-1 inhibited HIV replication, results from cell viability as determined by MTT assay showed no toxicity (Fig. 5C) and homologous recombination appeared to remain unaffected (data not shown).

## Discussion

While productive replication of HIV-1 occurs mainly in microglia and resident macrophages in the brain, astrocytes also provide a host for limited viral replication, expression of HIV-1 proteins and can be HIV-1 cellular reservoirs and important participants in neuropathogenesis [27]–[30]. Here, we report that the homology-directed DNA repair enzyme Rad51 regulates the activity of the HIV-1 LTR promoter in primary cultures of human astrocytes and acts in concert with the NF-κB pathway. Reporter experiments with LTR deletion mutants showed that both NF-κB p65 and Rad51 required the region spanning −120 to −80 of the LTR which contains the two copies of the κB binding site of the LTR. RNA interference for either NF-κB p65 or Rad51 inhibited the basal activity of the LTR. Similarly in the LTR-luciferase reporter cell line, TZM-bl, Rad51 increased LTR transcription while Rad51 siRNA decreased it.

As in many other published reports, LTR activity was increased by phorbol myristate acetate (PMA), which stimulates NF-κB translocation to the nucleus and by the histone deactetylase inhibitor trichostatin A (TSA), which stimulates acetylation of histones and nonhistone proteins including certain transcription factors such a NF-κB p65 [31], [32]. PMA results in enhanced Rad51 binding to the LTR and this is partially reversed by NF-κB p65 siRNA indicating that NF-κB p65 is responsible, at least in part, for recruiting Rad51 to the LTR. TSA also results in enhanced Rad51 binding to the LTR and this may be due to an increased binding of acetylated NF-κB p65 to the LTR as was we have reported for NF-κB p65 binding to the polyomavirus JC promoter [33]. A dominant negative mutant of IκBa, which inhibits the translocation of NF-κB p65 to the nucleus also inhibited the nuclear localization of Rad51 preventing LTR activation by Rad51 and, as expected, NF-κB p65 as measured by subcellular fractionation or immunofluorescence (Fig. 4). This suggests that the NF-κB pathway is involved in Rad51 nuclear translocation, perhaps involving its ability to form a complex with NF-κB p65 [19]. Note that this mechanism may be distinct from the mechanism involved after DNA damage where, unlike Fig. 4, Rad51 localizes to discrete isolated nuclear foci and involves BRCA2 [34]. In agreement with our data on the IκBa mutant, Wu et al [35] recently reported that the IκB kinase inhibitor BMS-345541 sensitizes MCF-7 breast cancer cells to ionizing radiation by selective inhibition of homologous recombinational repair of DNA double-strand breaks.

The importance of Rad51 in HIV infection is indicated by our results with HIV-1 infection of PBMCs and the Rad51 inhibitor RI-1, which significantly decreased infection with no toxic effects on viability. Thus, the interaction of Rad51 with the HIV-1 LTR that we have reported has an important function in the viral life cycle. Interestingly, Rad51 has been found to inhibit HIV-1 integration in vitro and the small molecule Rad51 stimulator RS-1 [36], which promotes active nucleofilament formation, inhibits HIV-1 replication in a HeLa P4 cell model [37]. This may mean that there is an optimum intracellular concentration of Rad51 for efficient replication of HIV-1 since Rad51 has a negative effect on integration but a positive effect on transcription. Alternatively, the effect of RI-1 on HIV-1 replication may depend on cell type in general and cell proliferation in particular since Rad51 expression fluctuates through the cell cycle reaching a maximum during S-phase [24]. We used PBMCs, which were cycling much more slowly (Fig. 5) whereas Cosnefroy et al [37] used proliferating HeLa P4 cells.

Taken together our data unravel a new level of complexity in the regulation of HIV-1 and an interconnection between its life cycle and the processes of DNA repair. The HIV-1 LTR is regulated by a variety of epigenetic and non-epigenetic mechanisms that serve to control the processes of transcription initiation and elongation and determine whether proviral DNA remains in a silent state of latency or reactivates to cause productive infection [38]. The finding that Rad51 is involved in this regulation suggests a connection with DNA repair since Rad51 is involved in the homologous recombination pathway of double-strand break DNA repair and is induced by DNA damage [20], [21]. Consistent with this role are reports from the literature that DNA damage can activate HIV [39]–[41] and induction of Rad51 is a possible mechanism whereby this occurs. Further, inhibition of the ATM kinase, which detects double-strand DNA breaks and activates the cellular DNA damage response, by small molecule inhibitor KU-559933 is capable of suppressing the replication of HIV-1 [42]. Since Rad51 acts with NF-κB p65 to regulate the HIV-1 LTR, it is of note that double-strand DNA breaks also activate NF-κB signaling [43], [44]. In addition, it has been found that activated NF-κB can induce the homologous recombination pathway of DNA repair, by interacting with the CtIP-BRCA1 complex, and accelerate Rad51 foci formation [45], [46]. Reciprocally, BRCA1 augments NF-κB activity by binding to the RelA domain of p65 [47]. In addition, it has recently been reported that transcriptionally active chromatin recruits homologous recombination [48] and this may be relevant to the connection between the role of Rad51 in stimulating HIV-1 transcription and its role in DNA repair.

In earlier studies, we demonstrated that infection of primary cultures of microglial cells with HIV-1 leads to an elevated level of Rad51 [19]. In corroboration with this data, we found that infection of primary cultures of PBMCs with HIV-1 can enhance levels of Rad51, only in those samples with low levels of Rad51 suggesting that activation of Rad51 by HIV-1 relates, in large part, to the basal level of this protein in the cells. The interplay between HIV-1 and Rad51 that we have described may have a role in regulating latent provirus in viral reservoirs and may also be an important positive feedback mechanism during active viral replication since we report here that HIV-1 infection reciprocally induces Rad51 levels. The mechanism of the induction of Rad51 by HIV-1 infection is unknown but we have found that expression of HIV-1 Tat elevates Rad51 protein levels resulting in increased repair of DNA double-strand breaks via homologous recombination [49]. Another possibility is that viral integration may activate DNA damage responses that induce Rad51, e.g., viral integration has been reported to activate DNA-dependent protein kinase and induce phosphorylation of p53 and histone H2AX [50].

In conclusion, our observations provide evidence for the involvement of DNA repair factors in the control of HIV-1 gene activation in astrocytes and thus offer a new avenue for the development of anti-viral therapeutics that target viral gene transcription in latently infected astrocytes and thereby manipulate this important viral reservoir.

## Materials and Methods

### Ethics Statement

Cultures of primary human fetal astrocytes were prepared from human fetal brain tissue obtained under approval of the Temple Institutional Review Board (IRB). The study was classified as exempt (category 4) by the IRB, and therefore, per IRB guidelines, a waiver of consent was approved and no informed consent was required or obtained.

### Plasmids and reagents

The HIV-1 LTR-luciferase reporter plasmid and its deletion mutants, pCMV-p65(NFκB) and pCMV-Rad51 (pcDNA6/Myc/His-A) expression plasmids have been previously described [19], [51], [52]. pCMV-IκB_mut_ was purchased from Clontech (Mountain View, CA USA). Human RelA ON-TARGETplus SMARTpool siRNA was purchased from Thermo Scientific (Waltham, MA USA). Human Rad51 siRNA was obtained from Santa Cruz Biotechnology (Santa Cruz, CA USA). Rad51 inhibitor RI-1 was kindly provided by Philip P. Connell, University of Chicago and has been previously described [25]. PMA and Trichostatin A were purchased from Sigma-Aldrich. The Rad51 inhibitor B02 was purchased from Millipore (Billerica, MA USA).

### Cell culture and transfection

Primary human fetal astrocytes were prepared from 16–18 week-old human fetal brain tissue (Advanced Biosecience Resources Inc., Almeda, CA USA) by modified procedure described previously [53], [54]. The TZM-bl is a derivative of HeLa cells that expresses high levels of CD4, CCR5 and CXCR4 and was previously designated JC53-bl (clone 13). TZM-bl cells contain reporter cassettes of luciferase and β-galactosidase that are each expressed from the HIV-1 LTR [55]. This reporter cell line was obtained through the NIH AIDS Research and Reference Reagent Program, Division of AIDS, NIAID, NIH and was contributed by Dr John C. Kappes, Dr Xiaoyun Wu and Tranzyme Inc. PBMCs were prepared from buffy coat fraction purchased from Biological Specialties Corporation (Colmar, PA) using Ficoll-Paque centrifugation. All transfections were performed using Lipofectamine 2000 (Life Technologies, Grand Island, NY) according to the manufacturer's protocol.

### Luciferase assay

Cells were lysed 48 h post-transfection using Passive Lysis Buffer (Promega, Madison, WI) and assayed with a Luciferase Reporter Gene Assay kit (Promega) according to the protocol of the manufacturer. Luciferase activity was normalized to total protein amount in each sample.

### Western blot analysis

Whole cell lysates were prepared with Passive Lysis Buffer (Promega), nuclear and cytoplasmic fraction were extracted using NE-PER reagent (Thermo Scientific) according to manufacturer's protocol. The primary antibodies used were anti-NF-κB p65 (C-20) and anti-GAPDH from Santa Cruz, anti-Rad51 D4B10 and anti-Lamin A/C from Cell Signaling, anti-α-tubulin clone B512 from Sigma-Aldrich.

### ChIP assay

TZM-bl cells were transfected using Lipofectamine 2000 reagent with 2.5 µg of pCMV-p65 and pCMV-Rad51 alone or together, with the total amount of DNA in each transfection being equilibrated with empty pCMV vector to 5 µg/100 mm dish, or with 150 nM human RelA siRNA and 150 nM hRad51 siRNA alone or together. The total amount of siRNA was equilibrated with non-target siRNA to concentration 300 nM. After 24 hours, cells were treated overnight with Trichostatin A (250 nM) and/or PMA (16.2 nM). The next morning cells were re-treated for 20 minutes and harvested according to the ChIP assay protocol (EMD Millipore, Billerica, MA). Immunoprecipitations were performed using lysates from 1×10^6^ cells per reaction with 1 µg of anti-NFκB p65 (C-20) rabbit polyclonal antibody, 1 µg of anti-Rad51 D4B10 rabbit monoclonal antibody or 1 µg of normal rabbit serum as a control. Extracted DNA was subjected to PCR using Fail Safe Kit buffer D, using primers for LTR -201/-3 sequence (forward: 5′-TAGAGTGGAGGTTTGACAGCCG-3′, reverse: 5′-GTACAGGCAAAAAGCAGCTGCT-3′), 30 cycles, 60°C annealing and resolved in 2% agarose gel. Data were normalized relative to signal from input DNA.

### Immunocytochemistry

Cells were plated in 4 well plastic chamber slides at a density of 2.5×10^4^/well and transfected the following day with pCMV-p65, pCMV-Rad51 together with pCMV-IκB_mut_ or empty pCMV vector plasmid. After 24 h, cells were treated with PMA (16.2 nM) overnight, fixed in 4% paraformaldehyde in PBS for 10 min, washed, permeabilized for 5 min with 0.1% TritonX-100, blocked for 30 min with 5% normal goat serum and incubated 3 h at 37°C with anti-NFkB p65 (rabbit polyclonal C-20, Santa Cruz) or anti-Rad51 (mouse mAb 14B4, GeneTex Inc.) at a 1∶100 dilution in PBS. Cells were then washed, incubated for 2 h with secondary FITC-conjugated goat anti-rabbit or anti-mouse secondary antibodies (as appropriate) at a 1∶200 dilution, washed, mounted with DAPI-containing mounting medium (VECTASHIELD, Vector Laboratories Inc. Burlingame, CA) and viewed by fluorescence microscopy.

### HIV-1 preparation and infection

HIV-1 SF_162_ was obtained through the NIH AIDS Research and Reference Reagent Program, Division of AIDS, NIAID, NIH: HIV-1 SF_162_ from Dr. Jay Levy. Infectious virus was prepared from PHA-induced PBMCs (5 µg/ml, 48 h) and tittered by TCID50 assay in TZM-bl cells. For experiments, freshly isolated PBMCs were induced with PHA for 48 h (5 µg/ml) then incubated with 10000 TCID50/10^6^ cells (MOI ∼0.01) of HIV-1 SF_162_ overnight. The next day, cells were washed 3 times with PBS and then incubated in the presence of 10 or 50 µM of Rad51 inhibitor (RI-1) for 3 days. Infection levels were quantified by p24 Gag ELISA (ABL Inc., Rockville, MD) of supernatants from infected cells. To asses cells viability upon treatment MTT assay was performed according to the manufacturer's protocol (Vybrant, Life Technologies).
